# Safety of oral naltrexone in HIV-positive men who have sex with men and transgender women with alcohol use disorder and initiating antiretroviral therapy

**DOI:** 10.1371/journal.pone.0228433

**Published:** 2020-03-05

**Authors:** Pedro Gonzales, Arielle Grieco, Edward White, Rona Ding, Rachel Bender Ignacio, Delia Pinto-Santini, Javier R. Lama, Frederick L. Altice, Ann Duerr

**Affiliations:** 1 Asociación Civil Impacta Salud y Educación, Lima, Peru; 2 University of Illinois at Chicago, School of Public Health, Chicago, IL, United States of America; 3 Vaccine and Infectious Disease, Fred Hutchinson Cancer Research Center, Seattle, WA, United States of America; 4 University of Washington, School of Medicine, Seattle, WA, United States of America; 5 Allergy and Infectious Diseases, Department of Medicine, University of Washington, Seattle, WA, United States of America; 6 Department of Internal Medicine, Yale School of Medicine, New Haven, CT, United States of America; 7 Department of Epidemiology of Microbial Diseases,Yale School of Public Health, New Haven, CT, United States of America; ARGENTINA

## Abstract

HIV disproportionately affects men who have sex with men (MSM) and transgender women (TW). These populations use alcohol more heavily than the general population, and alcohol use disorders (AUDs) are more prevalent among them. Naltrexone (NTX) has documented efficacy and safety as a medication-assisted therapy for AUD. Its use has not been well-examined in persons with HIV (PWH) newly initiating antiretroviral therapy (ART) where the possibility of hepatotoxicity may be increased when initating multiple new medications. This study assessed the safety of oral NTX treatment (50 mg daily) initiated concomitantly with antiretroviral therapy (ART) in a double-blind randomized placebo-controlled trial of NTX in MSM/TW in Lima, Peru among MSM and TW with AUD (AUDIT score ≥ 8). We analyzed adverse event data from ART-naïve participants (N = 155) who were randomized (2:1) to initiate ART plus NTX (N = 103) or ART plus placebo (N = 52). Participants were monitored for 24 weeks while taking ART plus NTX/placebo, followed by 24 weeks receiving ART alone. Over 48 weeks, 135 grade 2 or 3 adverse events were reported, resulting in 1.3 clinical adverse events per participant equally represented in both treatment and placebo arms. Two serious adverse events occurred among two participants receiving NTX; neither was attributed to the study medication. No significant differences were found in the proportion of subjects reporting any adverse events between treatment arms across all time-points. These results suggest NTX is safe in MSM/TW PWH with AUD newly initiating ART, as no excess of clinical adverse events or transaminase elevation was associated with NTX use.

## Introduction

HIV disproportionately affects men who have sex with men (MSM) and transgender women (TW) in Peru [1–3]. Use of alcohol and other drugs is common among people with HIV (PWH) [4,5], especially alcohol use disorders (AUDs) among MSM in Peru [6]. Alcohol use is associated with increased sexual risk behavior and decreased adherence to medication regimens, including antiretroviral therapy (ART), resulting in suboptimal health outcomes [7–12] and practices that can lead to onward transmission [13]. Use of alcohol or drugs can exacerbate the effects of HIV infection [14], therefore effective treatments for AUD in PWH are much needed. Similarly, treatment of HIV with ART may result in adverse clinical outcomes [15]. Medication-assisted therapy (MAT) is among the most effective strategies for treating AUD [16]. Naltrexone (NTX), a complete opioid antagonist, has documented efficacy and safety in treating AUD in the general population and superior to other MAT and counseling-based therapies [17,18]. Concerns over oral NTX's potential hepatoxicity have largely been allayed by randomized, placebo-controlled clinical trials [19], including in PWH [20] however, its safety and effectiveness in PWH receiving ART have been addressed in only one study, conducted with the participation of a cohort of US military veterans, 98% of which were male [21].

Recently, a randomized trial conducted in the US showed similar incidence of adverse events (AEs) in PWH who received extended release NTX by injection (XR-NTX) or placebo [22]. While XR-NTX may ensure more consistent adherence than may be achieved with oral NTX, and its efficacy and tolerability have been established for treatment of alcohol dependency, XR-NTX is not readily available outside the US, Europe and Australia [20,23,24].

Our study, conducted in Lima, Peru, evaluated the impact of oral NTX on ART adherence in a randomized trial among MSM and TW PWH whose responses to a standardized instrument indicated AUD. Written, informed consent was obtained following established standards, and protocols were approved by the Impacta Comite Institucional de Bioetica, Institutional Review Board Committee of the Fred Hutchinson Cancer Research Center, and the Yale University Human Investigation Committee.

We report here on the safety of NTX in this population, which was assessed by comparing reported AEs and laboratory abnormalities by treatment arm.

## Methods

### Study design

The AHORA study was a double-blind randomized placebo-controlled trial of oral NTX (50 mg/day) in HIV-infected MSM and TW who were initiating ART in Lima, Peru. The primary outcomes of this study were ART adherence and HIV virologic suppression, and are reported separately from this analysis of safety. AHORA was conducted during 2014 and 2015 and data were collected at in-person visits, including from clinical exams and laboratory test results, as well as by self-report using computer assisted self-interviews (CASI). Questionnaire items included the Alcohol Use Disorders Identification Test (AUDIT) [25]. Eligible participants: 1) were 18–64 years old, 2) had confirmed HIV infection, 3) self-identified as MSM or TW, 4) met criteria for AUD (indicated by AUDIT score ≥ 8) but had no evidence of alcohol withdrawal syndrome at screening, 5) had undergone no treatment for AUD in the prior 30 days, 6) had hepatic transaminases (ALT and AST) ≤ 3x the upper limit of normal within 90 days before enrollment, 7) had creatinine clearance ≥ 50 ml/min and 8) had not previously received ART. Participants were excluded if they: 1) had other current significant medical problems including chronic hepatitis B or cirrhosis or opportunistic infection, 2) reported or showed evidence of using opioids (including positive urine test for opioids), or 3) were incarcerated or anticipated hospitalization where pain medications would be required. Participants received care at one of two clinical research sites consistently throughout the course of the study.

Eligible participants were randomized 2:1 to oral NTX or placebo control arm. Randomization employed a computer-generated ‘minimization’ algorithm and adaptive randomization. Participants were followed for 24 weeks on NTX or placebo, and then for an additional 24 weeks after discontinuation of medication to assess whether any effects of NTX on ART adherence persisted during the post-intervention phase.

One hundred and fifty-nine participants (NTX n = 106, placebo n = 53) were initially enrolled and started on an oral ART regimen of co-formulated emtricitabine/tenofovir disoproxil fumarate/efavirenz (TDF/FTC/EFV) once daily (donated by Merck & Co). Participants with intolerance to efavirenz were switched to co-formulated TDF/FTC (donated by Gilead Sciences Inc), plus ritonavir-boosted lopinavir or atazanavir. Participants also received either 50mg oral NTX or matched placebo, per randomization, to be taken once daily. Nalerona^™^ (naltrexone) 50 mg and its placebo were obtained from Grunenthal Peruana SA. NTX or placebo adherence was assessed using e-cap^™^ (Information Mediary Corp, Ottawa, Canada), an electronic drug monitoring system, with download of data at monthly study visits. Adherence to NTX was calculated using the eCAPs monthly record of instances when a participant's medication bottle was opened.

Participants were followed for 24 weeks with periodic examinations, after which the NTX or placebo was stopped, and participants were followed on ART alone for an additional 24 weeks, to assess ART adherence and any AEs that occurred. At study end, all participants were transferred to the Peruvian Ministry of Health HIV care program for administration of clinical care and ART free of charge. All adverse events qualified as Treatment Emergent Adverse Events. Severity of AEs was graded in accordance the toxicity tables established by the Division of AIDS/NIH [26]. Data on demographics, recent use of alcohol and other drugs, and health status were collected by self-report using CASI at regularly scheduled visits. Laboratory monitoring included CD4 count, AST/ALT enzyme levels, HIV-1 RNA (viral load), and blood urea nitrogen (BUN) and creatinine levels. AST/ALT was measured at enrollment, biweekly intervals until week 8, then every four weeks until week 40. Many participants declined to provide information on two important indicators of socioeconomic status, health insurance status and income (16% and 57%, respectively); as such, these characteristics were excluded from analyses. All others (age, sex at birth, gender identity, sexual orientation, education, living situation) showed < 5% missing at baseline.

Level of AUD severity at baseline from the Alcohol Use Disorders Identification Test [27], were further divided into: hazardous drinking (score = 8–15), harmful drinking (16–19), and dependent drinking (score ≥ 20). Further alcohol assessments from CASI were collected at baseline, and weeks 12, 24, 36 and 48: number of drinks taken in the prior month, and whether the individual drank to the point of losing consciousness any time in the prior month prior. While formative research showed little stigma associated with alcohol use in the study population, initial self-reported data suggested social desirability bias. Therefore, we initiated collection of hair and blood specimens for tests of alcohol use biomarkers from a subset of participants. Alcohol use was assessed at week 24 using biomarkers detected by standard procedures in a commercial laboratory (US Drug Testing Labs, Des Plaines, IL). Ethylene glucuronide (EtG) in hair only in the last 88 participants recruited and phosphatidylethanol (PEth) was assessed in dried blood spots in the last 60 participants. Cut-offs for a positive result were 8 ng PEth/ml in blood and 8 pg EtG/mg in hair. For participants whose hair and DBS were collected at the same visit, alcohol use was scored as positive if either PEth or EtG was above the threshold. The Drug Abuse Screening Test (DAST-10) was included in the CASI questionnaire at baseline as well as at quarterly follow up visits up to week 48 [28]. It assessed use of drugs other than alcohol and tobacco. Scores were dichotomized as ≥ 3 (moderate and higher) and < 3 (low level and less). Data were collected on self-reported recent use of marijuana, cocaine powder, cocaine paste (a smokeable, unrefined paste made from coca leaves which is common in the Andes region), heroin, amphetamine, psychedelics in the previous year at baseline, and quarterly up to week 48.

Clinical and laboratory AEs were documented with participants’ reported date that symptoms started for clinical AEs and the date of specimen collection for laboratory AEs. Study clinicians documented their specific diagnoses of AEs and severity grade according to Division of AIDS (DAIDS) toxicity tables (coded here using Vers. 2.1, July 2017) [16]. For assessment of laboratory AEs, normal and upper-limit of normal (ULN) values were established by the participating laboratory in Lima. Concurrent diagnoses were documented as distinct AEs and received the same severity grade unless otherwise specified. Severity of AEs were graded from 1 (mild), 2 (moderate), 3 (severe), and 4 (potentially life-threatening). AEs were categorized as serious adverse events (SAEs) if they met DAIDS criteria for such, being: life threatening, requiring hospitalization, resulting in disability, requiring intervention to prevent permanent impairment or damage.

### Data analysis

Distributions of covariates were compared by treatment arm to assess the degree to which randomization was successful. Data management, cleaning, and analyses were conducted using Stata 14 (Stata/IC 14.2 for Mac, StataCorp) and R (R Studio Version 1.1463, R Studio) [29, 30]. We compared distribution of baseline characteristics between treatment arms using chi-square tests for binary variables, and Wilcoxon rank-sum tests for categorical variables (i.e., sexual orientation) and continuous variables with non-normal distribution (i.e., age). All statistical tests were conducted with alpha at 0.05.

Safety assessment analysis was conducted under an intent-to-treat (ITT) model. We compared differences in the proportions of individuals reporting any adverse event between treatment groups across study timepoints. In addition, number of adverse events by category and severity were compared by arm. A secondary analysis compared the number of AEs by level of adherence, between study arms.

## Results

**Fig 1:**
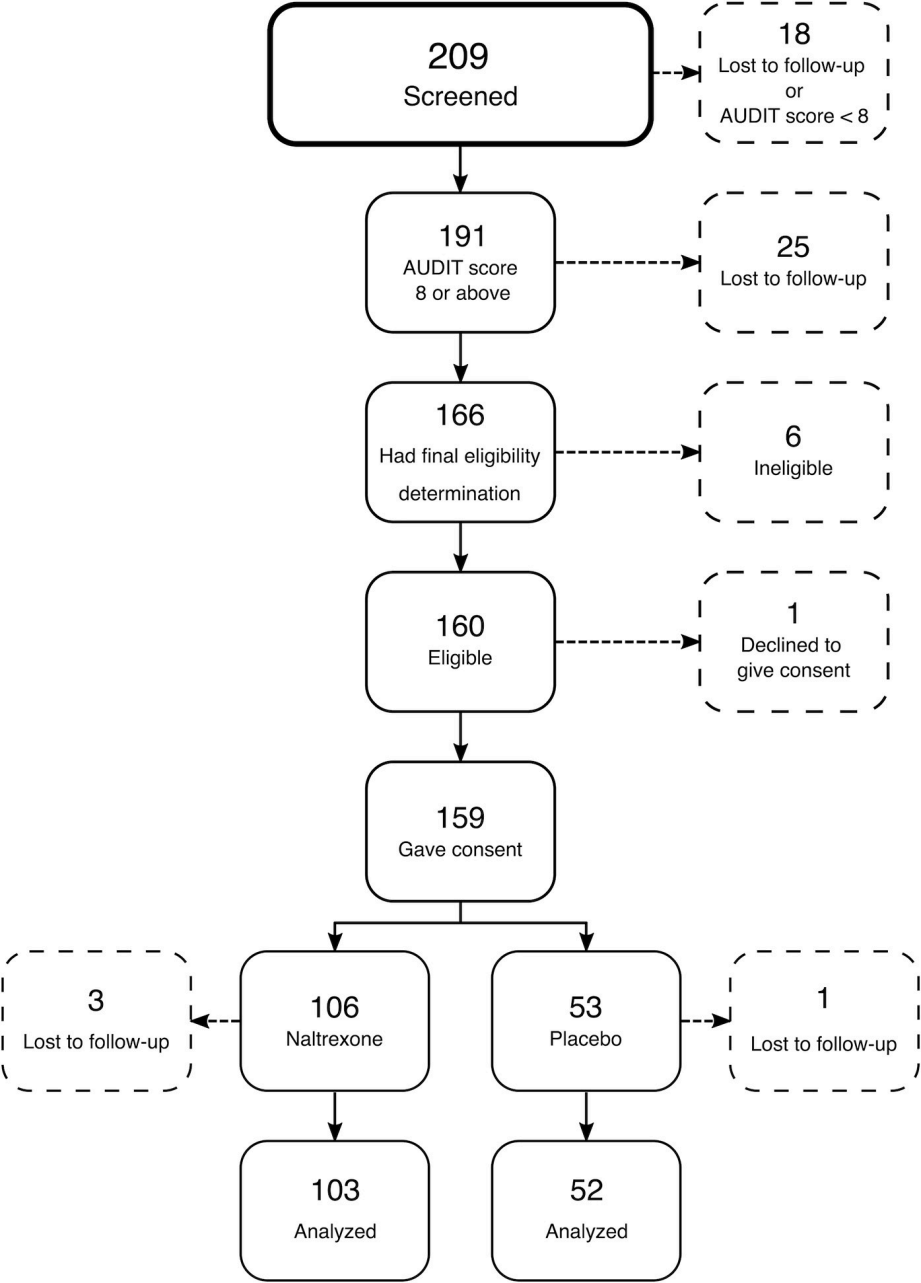
CONSORT diagram of AHORA study.

Three enrollees in the NTX arm and one in the placebo arm were lost to follow-up immediately after initial drug dispensation. We analyzed data from 103 participants receiving naltrexone and 52 participants receiving placebo (Fig 1).

The study population was young (median age = 26 years) and predominantly self-identified as homosexual. There were no notable differences in any covariates between arms with the exception of two. AUDIT scores were higher among those in the placebo arm, with 60% having scores above the overall median compared with 41% of those receiving NTX (p = 0.03). There was a non-significant, but appreciable difference in having the goal of stopping alcohol use permanently (p = 0.10) (Table 1).

**Table 1:** Baseline characteristics of participants by treatment arm.

Covariates	Overall n = 155		Treatment arm			
			Naltrexone (n = 103)		Placebo (n = 52)	
	Median (IQR) [Range]		Median (IQR) [Range]		Median (IQR) [Range]	
**Age**	26 (8) [18–50]		25 (10) [18–49]		26 (7) [18–50]	
**CD4 count**	355 (228) [13–1145]		358.5 (221) [13–1145]		343 (245.5) [17–775]	
**log10 HIV viral load**	4.98 (0.81) [2.12–6.86]		4.91 (0.81) [2.12–6.86]		5.05 (0.75) [3.39–6.14]	
**AST**	0.49 (0.22) [0.29–6.86]		0.51 (0.20) [0.29–1.63]		0.46 (0.24) [0.31–2.20]	
**ALT**	0.42 (0.31) [0.13–2.94]		0.44 (0.31) [0.13–2.26]		0.38 (0.29) [0.19–2.94]	
**Blood urea nitrogen**	11.5 (4.5) [3.8–28.7]		11.5 (4.5) [3.8–21.7]		11.6 (5.5) [6.2–28.7]	
**Sexual orientation (cisgender male only)**	N	%	N	%	N	%
Homosexual	108	76.1	72	76.6	36	75.0
Bisexual	29	20.4	19	20.2	10	20.8
Heterosexual	4	2.8	2	2.1	2	4.2
Other	1	0.7	1	1.1	0	-
**Gender**						
Cisgender male	142	91.6	94	91.3	48	92.3
Transgender female	11	7.1	9	8.7	2	3.8
**Education**						
Any tertiary	112	72.3	75	72.8	37	71.2
Up to secondary	41	26.5	28	27.2	13	25.0
**Income**						
Above national median	38	24.5	29	28.2	9	17.3
Below national median	30	19.4	19	18.5	11	21.2
Declined to answer	49	31.6	32	31.1	17	32.7
Did not know	46	23.2	23	22.3	13	25.0
**Living situation**						
Not living with a partner	137	88.4	94	91.3	43	82.7
Living with a male partner	13	8.4	7	6.8	6	11.5
Living with a female partner/wife	3	1.9	2	1.9	1	1.9
**Health insurance**						
Yes	41	26.5	26	25.2	15	28.9
No	89	57.4	64	62.1	25	48.1
Did not know	17	11.0	9	8.8	8	15.4
Declined to answer	6	3.9	4	3.9	2	3.9
**Alcohol use disorder**						
Hazardous drinking (AUDIT 8–15)	56	36.1	41	39.8	15	28.9
Harmful drinking (AUDIT 16–19)	33	21.3	22	21.4	11	21.2
Potential dependence (AUDIT≥20)	66	42.6	40	38.8	26	50.0
**Goal to stop drinking for some months**						
Yes	109	70.3	71	68.9	38	73.1
No	42	27.1	28	27.2	14	27.2
**Goal to stop drinking permanently**						
Yes	100	64.5	61	59.2	39	75.0
No	51	32.9	38	36.9	13	25.0
**Alcohol consumption to loss of consciousness (30 days)**						
Yes	31	20.0	19	18.5	12	23.1
No	120	77.4	80	77.7	40	77.4
**Drug use (past 30 days)**						
Yes	27	17.4	17	16.5	10	19.2
No	126	81.3	85	82.5	41	78.9

AUDIT = Alcohol Use Disorders Identification Test; Percentages may not equal 100% due to a small number of missing data.

We excluded from analysis eleven events for which participants reported onset of symptoms prior to provision of study medications. Over the 48 weeks of observation, 203 individual clinical AEs were documented on 194 occasions. Ninety-six (61%) participants presented with at least one clinical AE during the study; fifty-nine participants (39%) reported none. Of clinical AEs during the study period, 135 occurred among 63 of the 103 participants receiving NTX; 68 occurred among 31 the 52 participants receiving placebo. There were 1.31 clinical AEs per participant in both the NTX arm and placebo arm. Overall, 68 clinical AEs (34%) were categorized as Grade 1, 126 (62%) as Grade 2, and nine (4%) as Grade 3. There were no Grade 4 AEs, and no deaths. AE grade did not differ by study arm. The proportion of Grade 2 or Grade 3 clinical AEs was 64% among participants receiving NTX and was 72% among those receiving placebo (p = 0.23) (Table 2).

**Table 2:** Number and proportion of participants reporting any clinical AE or evidencing any laboratory AE, by treatment arm across study timepoints.

	Overall		Naltrexone arm		Placebo arm		
	n	%	n	%	n	%	p-value
**Clinical AEs**							
Cumulative to 24 weeks							
AE	81	52.3	54	52.4	27	51.9	0.95
No AE	74	47.7	49	47.6	25	48.1	
Cumulative to 48 weeks							
AE	96	61.9	65	63.1	31	59.6	0.67
No AE	59	38.1	38	36.9	21	40.4	
**Laboratory AEs**							
Cumulative to 24 weeks							
AE	31	20.0	18	17.5	13	25.0	0.27
No AE	124	80.0	85	82.5	39	75.0	
Cumulative to 48 weeks							
AE	37	23.9	23	22.3	14	26.9	0.53
No AE	118	76.1	80	77.7	38	73.1	

All tests performed were chi-square; n = 155

**Fig 2:**
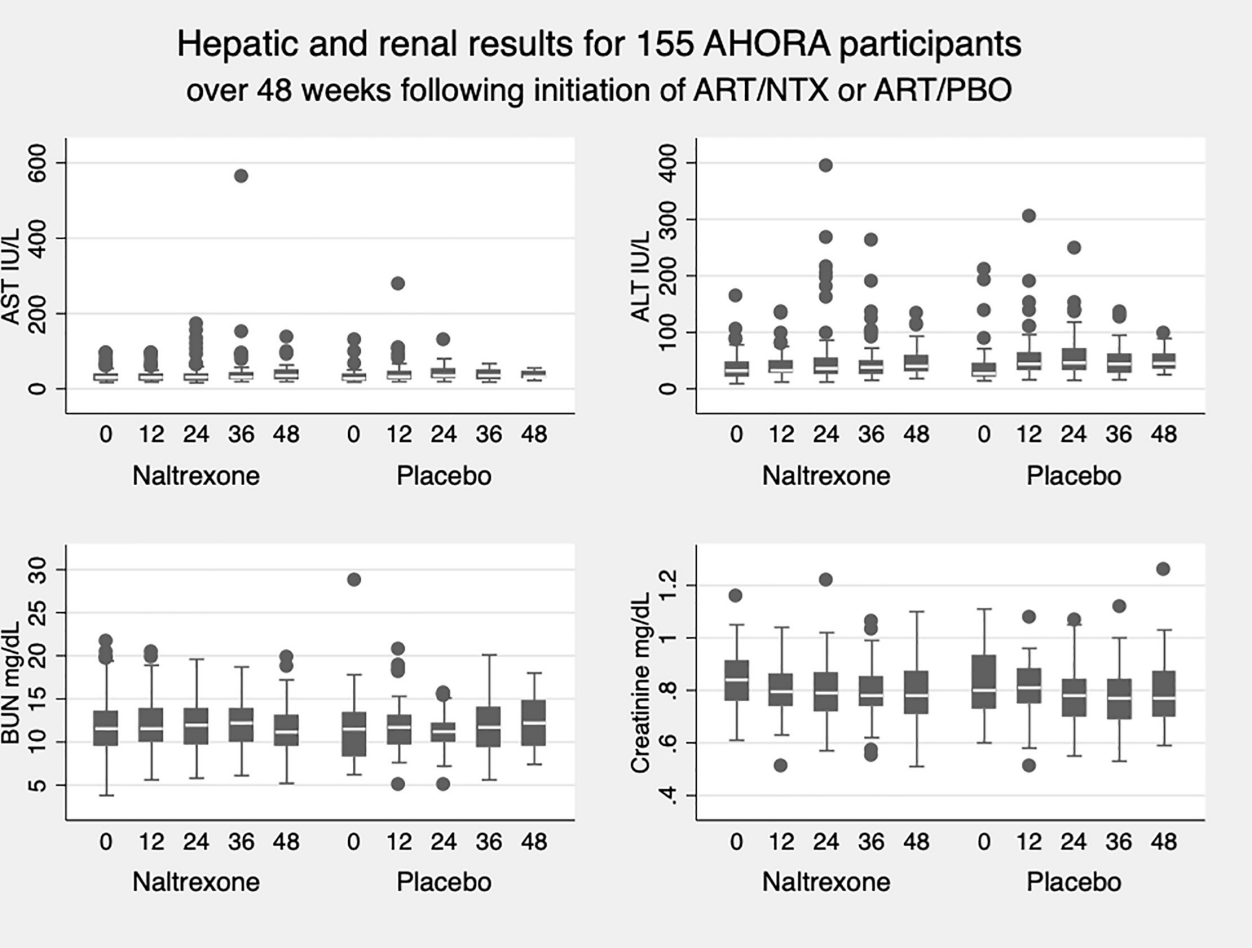
Hepatic and renal laboratory values.

All laboratory AEs were elevated levels of transaminases (alanine aminotransferase, ALT or SGPT; or aspartate aminotransferase, AST or SGOT). There were 60 such cases: 43 (72%) were Grade 1 (1.25–2.5 times ULN), 14 (23%) were Grade 2 (> 2.5–5.0 times ULN), and three (5%) were Grade 3 (>5.0–10 times ULN). In both study arms, levels of AST increased between enrollment and week 24, rising appreciably in the NTX arm (p = 0.10), and significantly in the placebo arm (p < 0.01) (Fig 2). ALT levels increased significantly in the NTX arm and the placebo arm between enrollment and week 24 (p < 0.01 in both). Over the 48 weeks of the study, 60 laboratory AEs occurred among 37 participants, 36 events among 23 individuals receiving NTX (n = 103) and 24 events among 14 individuals receiving placebo (n = 52) (Table 2). The ratio of transaminase elevations per participant in the NTX arm vs. the placebo arm was 0.8 (95% confidence interval [CI] 0.7–1.1). There were no instances of elevated creatine or BUN.

Adherence to NTX declined over time for participants in both study arms. At week 4, the proportion shown by eCaps to be taking < 25% of prescribed pills over the previous 30 days was 15% among those receiving NTX and 9% among those receiving placebo. At week 24, the proportion taking < 25% of pills was 46% among those receiving NTX and 43% among those receiving placebo (p = 0.74). A separate measure of adherence was also calculated; the total number of prescribed daily doses that had been taken, according to eCaps, divided by the number of days that the participant was active in the study.

Clinical AEs were attributed to NTX or ART based on previously reported clinical experience and local physician report. There were 54 attributable AEs (AAEs) among 41 study participants; 39 occurred among 29 participants on NTX and 13 occurred among 12 participants on placebo (Table 3).

**Table 3:** Cumulative clinical adverse events attributable to study drug (naltrexone or ART) by WHO preferred term, by treatment group, at treatment timepoints.

	During NTX/PBO (0–24 weeks)			Total Study (0–48 weeks)		
Clinical AEs	OverallN (%)	NTX n (%)	PBO n (%)	Overall N (%)	NTX n (%)	PBO n (%)
*Total*	*50 (100)*	*41 (100)*	*9 (100)*	*54 (100)*	*44 (100)*	*10 (100)*
Allergic dermatitis	2 (4.0)	2 (4.9)	0 (-)	2 (3.7)	2 (4.5)	0 (-)
Anxiety	1 (2.0)	1 (2.4)	0 (-)	1 (1.9)	1 (2.3)	0 (-)
Dizziness	11(22.0)	11 (22.4)	0 (-)	11 (20.3)	11 (25.0)	0 (-)
Gastritis	3 (3.0)	2 (4.9)	1 (11.1)	3 (5.6)	2 (4.5)	1 (10.0)
Gynecomastia	0 (-)	0 (-)	0 (-)	1 (1.9)	1 (2.3)	0 (-)
Headache	2 (4.0)	2 (4.9)	0 (-)	2 (3.7)	2 (4.5)	1 (10.0)
Hepatitis	0 (-)	0 (-)	0 (-)	1 (1.9)	1 (2.3)	0 (-)
Nausea/vomiting	9 (18.0)	9 (22.0)	0 (-)	9 (16.7)	8 (18.2)	0 (-)
Rash	21 (44.0)	13 (44.8)	8 (88.9)	21 (41.2)	13 (29.5)	8 (80.0)
Somnolence	1 (2.0)	1 (2.4)	0 (-)	1 (1.9)	1 (2.3)	0 (-)

**Fig 3:**
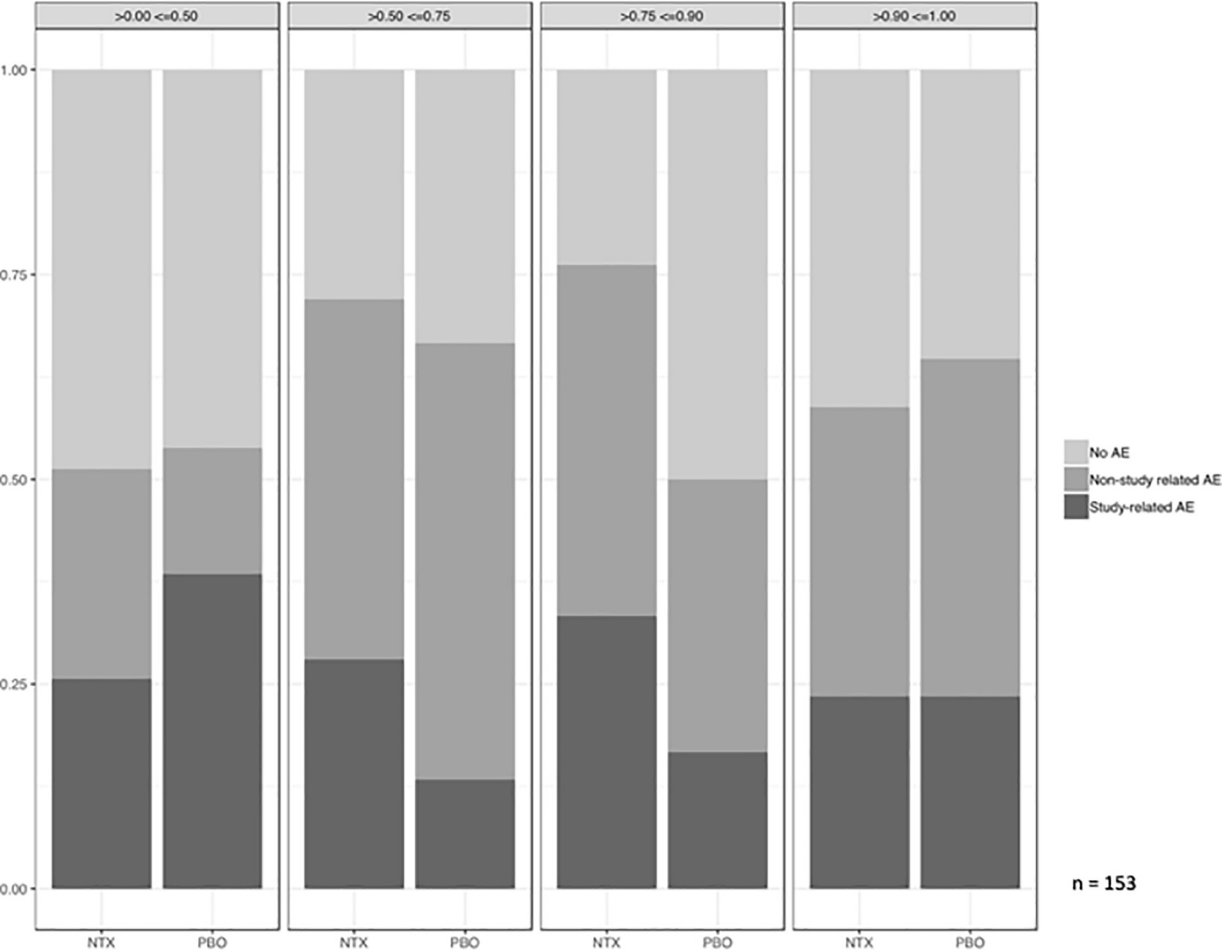
Adverse events, attributed to study drug, and unattributed to study drug, by treatment arm and adherence.

The ratio of AAEs per participant in the NTX arm vs. the placebo arm was 1.5 (95% CI 0.8–3.1). The distribution of participants experiencing AAEs, unattributable AEs (UAEs), and no AE did not differ by study drug adherence level (p = 0.86) (Fig 3). Similar proportions of AAEs of Grades 2 or 3 occurred among AEs in the NTX arm and placebo arm (62% and 68% respectively, p = 0.38). Two AEs, both in the NTX arm, were classified as SAEs. Study clinicians deemed that both were unrelated to study drugs (suicidal ideation, bone fracture).

Of 60 participants providing dried blood spots for analysis for PEth at week 24, 42 (70%) tested positive, indicating recent alcohol use. Of 88 participants providing hair specimens for analysis for EtG, valid results were obtained for 58. Of these, 29 had positive results, indicating moderate to high alcohol use in the past month [31]. Of 82 participants with valid results for either EtG or PEth, 49 (60%) indicated recent alcohol use. Of those whose biomarkers indicated recent alcohol use, 34 (69%) had a clinical AE during the course of the study. Among the 33 participants without indication of recent alcohol use, 21 (64%) had a clinical AE by 48 weeks; the difference was non-significant.

## Discussion

To our knowledge, this is the first study to examine adverse events in PWH initating both ART and NTX simultaneously. Moreover, this study was assessed within a placebo-controlled trial where detection bias of an adverse event might be differentially assigned as attributable. This has important implications because PWH have high levels of AUD and clinicians are often reluctant to start HIV treatment in patients with substance use disorders [32]. Key findings here suggest that there is no increased risk for adverse events associated with NTX compared to placebo in HIV-infected MSM/TW with AUD who concurrently initiated ART. The current study was successful in its recruitment of participants meeting the eligibility criteria as well as randomization of subjects. No differences in dropout rate between arms and no systematic missingness of data were found. Our findings are consistent with those of Tetrault et al., who found that oral NTX did not increase hepatic enzymes in PWH, but many in that observational study of veterans were already on ART or for those who initated it, the ART regimens were variable and could differentially contribute to adverse events and that patients receiving NTX were treated based on selection by clinicians [21]. Moreover, this study is consistent with a placebo-controlled trial of extended-release naltrexone (XR-NTX) administered to prisoners on ART before release from prison where there were no attributable excess AEs in the XR-NTX arm relative to placebo and in this study, a large proportion of PWH were co-infected with HCV [20]. Thus, our study indicates, that it is not only safe to prescribe ART and NTX concurrently, but to initiate them concurrently.

The primary limitation in these analyses is small sample size (n = 155). The sample size was chosen to provide adequate power to assess effects on ART adherence but provided limited power to detect differences in rare safety outcomes. Moreover, we randomized 2:1 to ensure we had a sufficient number of participants to measure adverse consequences. Despite this strategy, however, we were unable to more robustly explore infrequent safety outcomes, or examine defined subgroups who may be vulnerable to NTX-related AEs when taken concurrently with ART. Since both ART and NTX/placebo were initiated concurrently, the relationship of AEs to specific medications is difficult to ascertain.

Future analyses with increased sample sizes can address the possibility of rare adverse events. Additionally, outcomes related to treatment of AUD (reported separately) can help comprehensively weigh the risks and benefits of jointly initiating NTX and ART treatment.

## Conclusions

Concomitant initation of ART and treatment for AUD using NTX can be safely deployed in PWH and AUD. In the future, larger ecological studies of NTX and ART will need to explore adverse consequences from treatment, but alignment between preliminary data from this placebo-controlled RCT with other studies suggest that it is safe.
